# Relative Burdens of the COVID-19, Malaria, Tuberculosis, and HIV/AIDS Epidemics in Sub-Saharan Africa

**DOI:** 10.4269/ajtmh.21-0899

**Published:** 2021-10-11

**Authors:** David Bell, Kristian Schultz Hansen

**Affiliations:** ^1^ Independent Consultant, Issaquah, Washington;; ^2^ Department of Public Health and Centre for Health Economics and Policy, University of Copenhagen, Copenhagen, Denmark;; ^3^ The National Research Center of Working Environment, Copenhagen, Denmark

## Abstract

COVID-19 has had considerable global impact; however, in sub-Saharan Africa, it is one of several infectious disease priorities. Prioritization is normally guided by disease burden, but the highly age-dependent nature of COVID-19 and that of other infectious diseases make comparisons challenging unless considered through metrics that incorporate life-years lost and time lived with adverse health. Therefore, we compared the 2020 mortality and disability-adjusted life-years (DALYs) lost estimates for malaria, tuberculosis, and HIV/AIDS in sub-Saharan African populations with more than 12 months of COVID-19 burden (until the end of March 2021) by applying known age-related mortality to United Nations estimates of the age structure. We further compared exacerbations of disease burden predicted from the COVID-19 public health response. Data were derived from public sources and predicted exacerbations were derived from those published by international agencies. For sub-Saharan African populations north of South Africa, the estimated recorded COVID-19 DALYs lost in 2020 were 3.7%, 2.3%, and 2.4% of those for tuberculosis, HIV/AIDS, and malaria, respectively. Predicted exacerbations of these diseases were greater than the estimated COVID-19 burden. Including South Africa and Lesotho, COVID-19 DALYs lost were < 12% of those for other compared diseases; furthermore, the mortality of compared diseases were dominant in all age groups younger than 65 years. This analysis suggests the relatively low impact of COVID-19. Although all four epidemics continue, tuberculosis, HIV/AIDS, and malaria remain far greater health priorities based on their disease burdens. Therefore, resource diversion to COVID-19 poses a high risk of increasing the overall disease burden and causing net harm, thereby further increasing global inequities in health and life expectancy.

## INTRODUCTION

COVID-19 has massively impacted life and society in sub-Saharan Africa, as it has elsewhere. Despite relatively low COVID-19 mortality rates in most countries of sub-Saharan Africa, aspects of the lockdown responses, including business and school closures and restricted health service access introduced in the early days of the pandemic, continue in various forms.^1^ In South Africa, infection with the virus itself has significantly impacted health, with 28,469 deaths attributed by the end of 2020.^1^ However, the younger populations to the north have recorded far lower mortality rates.^2^ Public health interventions must be tailored to address such variations. This requires realistic metrics for disease burden that consider the characteristics of the population and the individual impacted by disease.^3^

Time-based measures such as disability-adjusted life-years (DALYs) that incorporate loss of healthy life because of premature death and time lived with less than optimal health have the potential to better represent the full impact of disease, as opposed to mortality alone. This is of particular relevance when the disease being measured is highly age-related. This is not a reflection of the value of human life; all lives are considered of equal value. The use of DALYs lost serves as an important guide for resource allocation to ensure that the greatest health impact is achieved. A young child dying from pneumonia is clearly expected to lose more potential life-years than an 80-year-old dying from the same; therefore, interventions based on metrics that prioritize childhood pneumonia will achieve a greater overall impact. In resource-constrained sub-Saharan Africa, metrics of disease burden are of particular importance. Malaria imparts a disproportionate burden on DALYs lost because most mortality occurs before age 5 years. HIV/AIDS causes long periods of severe illness and premature death, primarily among young and middle-aged adults, leading to significant life-years lost and extended time lost because of less than optimal health.

COVID-19 is characterized by its strong association with advanced age, with a mean age at the time of death similar to that for all-cause mortality in many countries.^5^^,^^6^ However, reporting of the burden of COVID-19 has generally ignored age at the time of death and instead centered on comparisons of total mortality alone. Estimates based on total mortality also ignore the strong association of death from COVID-19 with pre-existing morbidities, which further reduce expected life-years lost.^6^

Low COVID-19-associated mortality among sub-Saharan African populations is at least partially predicted by young age structure.^5^^–^^7^ Lifestyle factors may be protective through a lower prevalence of major comorbidities,^8^^–^^10^ higher vitamin D levels, and broad antigen exposure leading to prior nonspecific T-cell immunity.^11^^–^^13^ Comparisons of lockdown severity suggest that more restrictive measures have had a limited additional impact on reducing COVID-19 mortality.^14^^–^^16^ However, as with many public health responses, lockdown responses are not without cost. Predicted exacerbations of high-burden diseases including malaria, HIV/AIDS, and tuberculosis particularly impact children and younger adults.^17^^–^^19^ Broader impacts of reduced food security and interruption of vaccination will have far-reaching health consequences,^20^^,^^21^ and the loss of family income and reductions in the national gross domestic product will impede the capacity to respond.^22^

Although lockdowns may be easing, proposals for continent-wide mass vaccination under the COVAX mechanism will raise new costs and divert resources, and the urgency of developing a good public health policy that appropriately prioritizes management of diseases including COVID-19, based on their relative burdens, is no less urgent.^23^ Therefore, we compared the disease burdens of COVID-19 and the three pre-existing major infectious disease epidemics of sub-Saharan African countries,^24^ with and without South Africa and Lesotho, to estimate the relative burden of COVID-19 in relation to these other epidemics.

## METHODS

### Health indicators and geography.

The health indicators used for the analyses were the number of deaths and disability-adjusted life-years (DALYs) lost by age group caused by COVID-19 and three major diseases, malaria, HIV/AIDS, and tuberculosis. Published data were sourced for the analyses. To calculate DALYs lost caused by these diseases at the population level, the estimates of numbers of deaths by age and the numbers of nonfatal episodes of illness by age in 2020 were required. The geographical area assessed was sub-Saharan Africa, excluding the five countries bordering the Mediterranean Sea. Estimates with and without South Africa and Lesotho were included because these two countries have very different burdens from the four diseases and differing demographics.

### COVID-19 mortality and DALYs lost.

Because COVID-19 cases were reported in Africa in February 2020, and widely spread by late March, all recorded COVID-19 deaths were included until March 31, 2021, to include slightly more than 12 months of reporting. According to the Africa CDC, there were 77,463 reported COVID-19 deaths in sub-Saharan Africa by March 2021, and 24,302 COVID-19 deaths when South Africa and Lesotho were excluded.^2^ These total reported COVID-19 deaths were allocated across age groups and used to estimate the number of COVID-19 infections by applying the following method and assumptions. Using COVID-19 death data and seroprevalence survey data from 45 mainly European countries, the infection fatality ratio by age was estimated.^25^ Assuming that these estimated infection fatality ratios also represented the sub-Saharan African situation, and further assuming a constant share of infection across age groups, the numbers of infections and deaths were inferred for a sub-Saharan Africa population size according to the United Nations population estimate for 2020 and compatible with a total number of 77,463 (24,302) COVID-19 deaths (see Supplemental Table S1 in the Supplemental Materials).^26^ These estimated numbers of deaths and infections by age group were inserted in the standard DALY formula used for calculating the burden of disease with no discounting of the future life-years and without the age-weighting function.^27^ To calculate life-years lost, the standard life expectancies by age from the Global Burden of Disease Study 2019 were used,^28^ and the reference life table was downloaded from the Institute of Health Metrics and Evaluation website.^29^ The nonfatal COVID-19 infections were assumed to be mild, with a 2-week duration, and a disability weight of 0.051 corresponding to the weight attached to a moderate to severe upper respiratory infection was used.^28^

### Mortality and DALYs lost because of HIV/AIDS, tuberculosis, and malaria.

The numbers of deaths and DALYs lost because of HIV/AIDS and tuberculosis by age group for 2019 were extracted from the Global Burden of Disease Study 2019 results.^29^ These estimates were updated to 2020 by assuming a growth from 2019 to 2020 corresponding to the annual population growth rate in sub-Saharan Africa. Population growth rates were estimated by age group using population estimates from 2015 and 2020.^26^ Cases of combined HIV/AIDS and tuberculosis are considered as HIV/AIDS only and are not included in the tuberculosis burden calculations.

The total number of deaths and nonfatal illness episodes caused by malaria in sub-Saharan Africa were obtained from the WHO estimates for 2019.^4^ However, the published numbers were not available by age group. It was assumed that the deaths and illness periods followed the same distribution across age groups as malaria deaths estimated by the Institute of Health Metrics and Evaluation.^29^ The numbers of malaria deaths and illness periods by age in sub-Saharan Africa (with and without South Africa and Lesotho) were translated into DALYs lost by applying the same method as described including the assumption that a nonfatal malaria infection lasted 2 weeks and with a disability weight of 0.051.

### Predicted exacerbations of HIV/AIDS, tuberculosis, and malaria.

Predicted exacerbations of the three compared diseases accrued from 2020 lockdown responses were derived from modeling published by the WHO, StopTB Partnership, and The Global Fund, simulating effects on transmission of reduced healthcare access and, for malaria, reduced vector control.^17^^–^^19^

### Patient and public involvement.

There was no patient involvement in this study. All data were obtained from publicly available sources.

### Ethics approval.

The analysis was based entirely on publicly available data. No specific ethical approval was required.

## RESULTS

**Figure 1.**
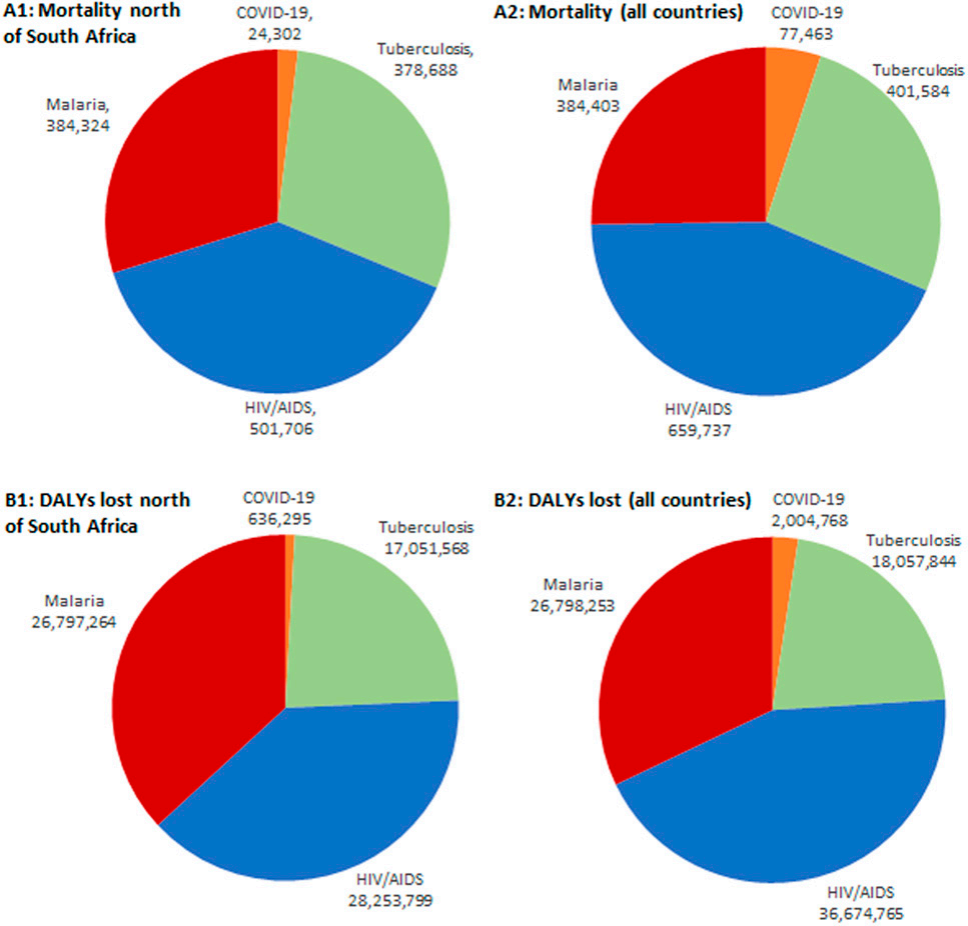
Comparison of baseline mortality and disease burdens (disability-adjusted life-years [DALYs] lost) predicted for the 12 months of 2020 for malaria, tuberculosis, and HIV/AIDS (impact before lockdown) and until March 31, 2021, for COVID-19 in sub-Saharan Africa. (**A1**) Mortality for sub-Saharan countries north of South Africa and Lesotho. (**A2**) Mortality for all sub-Saharan countries. (**B1**) DALYs lost for sub-Saharan countries north of South Africa and Lesotho. (**B2**) DALYs lost for all sub-Saharan countries. This figure appears in color at www.ajtmh.org.

Recorded COVID-19 mortality comprised 6.4%, 4.8%, and 6.3% of the mortality caused by tuberculosis, HIV/AIDS, and malaria, respectively, in sub-Saharan Africa north of South Africa and Lesotho. Regarding DALYs lost, the COVID-19 burden comprised 3.7%, 2.3%, and 2.4% of that estimated for tuberculosis, HIV/AIDS, and malaria, respectively (Figure 1). Including South Africa and Lesotho, the recorded COVID-19 mortality rates were 19.2%, 11.7%, and 20.2% of the mortality rates attributable to tuberculosis, HIV/AIDS, and malaria, respectively. Regarding DALYs lost, the COVID-19 burden comprised 11.1%, 5.5%, and 7.5% of DALYs lost to tuberculosis, HIV/AIDS, and malaria, respectively (Figure 1).

**Figure 2.**
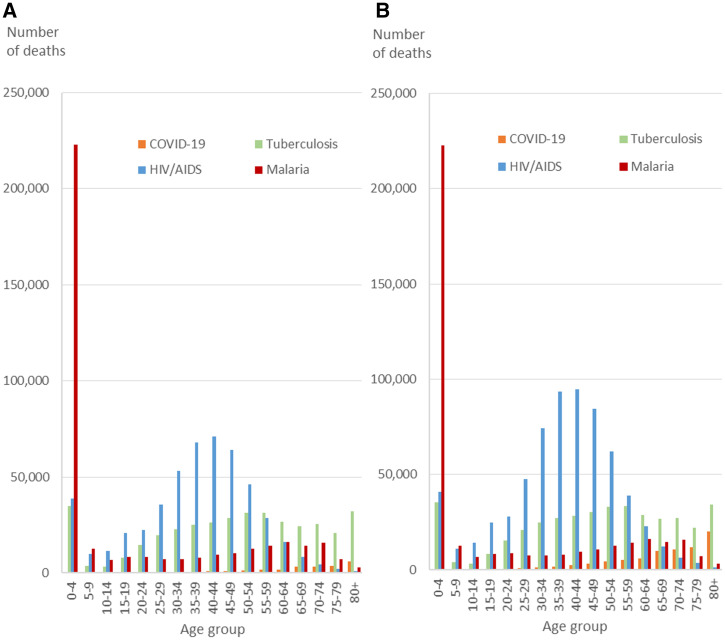
Mortality predicted for the 12 months of 2020 for malaria, tuberculosis, and HIV/AIDs (impact before lockdown) and until March 31, 2021, for COVID-19 in sub-Saharan Africa. (**A**) Mortality for sub-Saharan countries north of South Africa and Lesotho. (**B**) Mortality for all sub-Saharan countries. This figure appears in color at www.ajtmh.org.

Tuberculosis mortality was never dominated by COVID-19 in any age group, whereas HIV/AIDS was only dominated in the age group of 70 to 74 years, and malaria dominated in the age group older than 75 to 79 years in sub-Saharan Africa north of South Africa and Lesotho (Figure 2). Including South Africa and Lesotho, tuberculosis, HIV/AIDS, and malaria individually dominated COVID-19 mortality until age 65 to 69 years, after which COVID-19 dominated HIV/AIDS. Furthermore, malaria also dominated the groups 75 years and older (Figure 2).

Accrued COVID-19 DALYs lost until March 31, 2021, were lower than the lower-limit estimates of exacerbation for malaria, tuberculosis, and HIV/AIDS (increase over baseline) predicted because of the COVID-19 public health response in sub-Saharan Africa north of South Africa, and lower than those for tuberculosis and HIV/AIDS for sub-Saharan Africa as a whole. If we assume significant under-reporting of COVID-19 mortality and arbitrarily multiply by a factor of 10, then the DALYs lost because of COVID-19 only dominate the lower predicted exacerbations of the malaria burden in sub-Saharan Africa north of South Africa. It remains lower than all baseline disease burdens for malaria and HIV/AIDs in sub-Saharan Africa as a whole and all compared diseases when South Africa and Lesotho are excluded (Table 1).

**Table 1:** Comparisons of recorded disease burden (DALYs lost) from COVID-19 in sub-Saharan Africa until March 31, 2021, compared with predicted exacerbations for malaria, tuberculosis, and HIV/AIDS from the impact of the COVID-19 public health response

		DALYs lost			
		COVID-19	Malaria	Tuberculosis	HIV/AIDS
Sub-Saharan Africa	Baseline	2,004,768	26,798,253	18,057,844	36,674,765
	Exacerbation, low		1,901,866	10,769,549	15,579,790
	Exacerbation, high	20,047,677*	26,783,055	14,095,787	24,474,183
Sub-Saharan Africa excluding South Africa and Lesotho	Baseline	636,295	26,797,264	17,051,568	28,253,799
	Exacerbation, low		1,907,695	10,169,414	12,002,484
	Exacerbation, high	6,362,955*	26,793,081	13,310,297	18,854,617

DALYs = disability-adjusted life-years.

* COVID-19 mortality arbitrarily multiplied by 10 to simulate gross under-reporting.

## DISCUSSION

These comparisons emphasize the relatively low disease burden that COVID-19 is exerting on sub-Saharan African populations, with the pre-existing epidemics of malaria, tuberculosis, and HIV/AIDS all greatly dominating in life-years impacted, and with mortality dominating in all except the elderly. The use of DALYs lost to assess the relative disease burden follows well-accepted practice,^30^^,^^31^ but it is relatively unusual for assessments of COVID-19. It is unclear why this standard public health metric, or the related metric of quality-adjusted life-years, has not been widely applied to a disease with such a characteristic age-dependent impact. An appropriate assessment of burden is of extreme importance because decisions regarding resources to be allocated to vaccination as well as the imposition of recurrent lockdowns and other cost-bearing responses are made globally. Mass vaccination of the sub-Saharan African population against COVID-19, as is advocated in some areas,^23^ will draw essential resources from interventions aimed at health problems with far greater burdens through the diversion of financial resources and personnel on the ground. Such a wide-scale vaccination intervention has never been attempted before, and the implications for already overstretched health services will be significant. To ensure equity in health care, a comprehensive economic evaluation comparing costs and effects of interventions against all four epidemics, including a cost-effectiveness analysis, is urgently needed.

This data analysis had some limitations. COVID-19 mortality reporting in sub-Saharan Africa is incomplete, although low mortality is predicted by the population age structure and lower prevalence of major comorbidities, including obesity.^5^^,^^7^^,^^32^ Other lifestyle factors and prior immunity may also be protective.^10^^,^^12^^,^^33^^,^^34^ Because of the lack of strong local data regarding age-related mortality, we assume that this will reflect rates found elsewhere.^25^ Lack of transmission appears an unlikely explanation for low recorded mortality because high seroprevalence has been recorded in various sub-Saharan African settings.^33^^–^^39^ Although the higher mortality of COVID-19 in South Africa could be partially explained by higher reporting rates, South Africa also has higher rates of known mortality risk factors.^40^ Evidence of very high asymptomatic infection^39^ and the level of testing occurring (868,823 tests for 333 deaths in Uganda alone by February 23, 2021)^41^ suggest that the relatively low recorded mortality in most countries reflects reality similar to that in much of Asia.^42^

The relative burden of COVID-19 in 2020 is also subject to the first cases being reported in March in most of these populations;^42^ therefore, it may not have spread to all populations within the first few months of recording. However, the total mortality rate remains low across the continent at time of writing.^42^ Although significant increases have occurred in countries bordering South Africa from mid 2021, the reported mortality in the more populous countries to the north has remained low by global comparisons.^42^ The bulk of the COVID-19 burden may have been accrued during the approximately 12 months of total recording time used here.

DALYs lost to COVID-19 morbidity as estimated during this work do not account for postviral syndromes (e.g., “long covid”). These have limited prevalence and may be lesser (less severe illness) in younger African populations,^43^ but this is still unclear and will add somewhat to the COVID-19 burden. Conversely, the age-based nature of DALYs lost applied to COVID-19 does not consider the high prevalence of life-shortening comorbidities associated with these cases,^6^ which will, in turn, lead to an overestimation of the actual life-years lost. Even assuming 90% underestimation of COVID-19 mortality here, malaria and HIV/AIDS disease burdens still dominate COVID-19, as do most upper estimates of exacerbation of these through the COVID-19 public health response.

When comparing the impact of COVID-19 and other health burdens, we considered just three diseases. The broad impact of malnutrition, reduced educational attainment (closed schools), and damage to local and national economies will have major long-term impacts on populations and societal health.^20^^–^^22^^,^^44^ As a greater proportion of the population achieves postinfection immunity,^35^^–^^37^^,^^41^^,^^45^^,^^46^ the COVID-19 burden is likely to further decrease, and the cost-effectiveness of response interventions may decrease further. Therefore, it is imperative that cost-effectiveness analyses of further public health responses to COVID-19 in these sub-Saharan African populations are tailored to local need based on realistic metrics that reflect the impact of COVID-19 relative to other high-burden diseases. These analyses should also consider any potential negative impacts of these responses, including reduced health system access and resource diversion that may result from severe lockdowns or mass vaccination. Failure to address these issues will risk increasing health inequities rather than reducing them.
